# Angiotensin-(1–7) treatment blocks lipopolysaccharide-induced organ damage, platelet dysfunction, and IL-6 and nitric oxide production in rats

**DOI:** 10.1038/s41598-020-79902-x

**Published:** 2021-01-12

**Authors:** Hsin-Jung Tsai, Chih-Chin Shih, Kuang-Yi Chang, Mei-Hui Liao, Wen-Jinn Liaw, Chin-Chen Wu, Cheng-Ming Tsao

**Affiliations:** 1 Department of Anesthesiology, Taipei Veterans General Hospital, No. 201, Sec. 2, Shipai Rd., Beitou District, Taipei, 112 Taiwan; 2 Department of Pharmacology, National Defense Medical Center, Taipei, Taiwan; 3 Department of Anesthesiology, National Yang-Ming University, Taipei, Taiwan; 4 Department of Nursing, Oriental Institute of Technology, New Taipei City, Taiwan; 5 Department of Anesthesiology, Chung Shan Medical University and Hospital, Taichung, Taiwan; 6 Department of Anesthesiology, National Defense Medical Center, Taipei, Taiwan

**Keywords:** Bacterial infection, Sepsis

## Abstract

Sepsis can lead to shock, multiple organ failure, and even death. Platelets play an active role in the pathogenesis of sepsis-induced multiple organ failure. Angiotensin (Ang)-(1–7), a biologically active peptide, counteracts various effects of Ang II and attenuates inflammatory responses, reactive oxygen species production, and apoptosis. We evaluated the effects of Ang-(1–7) on organ injury and platelet dysfunction in rats with endotoxaemia. We treated male Wistar rats with saline or lipopolysaccharide (LPS, 10 mg, intravenously) then Ang-(1–7) (1 mg/kg, intravenous infusion for 3 h beginning 30 min after LPS administration). We analysed several haemodynamic, biochemical, and inflammatory parameters, as well as platelet counts and aggregation. Ang-(1–7) improved hypotension and organ dysfunction, and attenuated plasma interleukin-6, chemokines and nitric oxide production in rats after LPS administration. The LPS-induced reduction in platelet aggregation, but not the decreased platelet count, was restored after Ang-(1–7) treatment. The protein expression of iNOS and IκB, but not phosphorylated ERK1/2 and p38, was diminished in Ang-(1–7)-treated LPS rats. The histological changes in liver and lung were significantly attenuated in Ang-(1–7)-treated LPS rats. Our results suggest that Ang-(1–7) ameliorates endotoxaemic-induced organ injury and platelet dysfunction, likely through the inhibition of the inflammatory response and nitric oxide production.

## Introduction

Sepsis is defined as life-threatening organ dysfunction induced by a dysregulated host response to the invasion of microbes^1^. The pathophysiology of sepsis-induced multiple organ dysfunction is characterised by an early pro-inflammatory state, followed by immune system dysfunction, reactive oxygen species (ROS) production, immunothrombosis, and resultant disseminated intravascular coagulation (DIC), all of which are involved in various complicated illnesses^2,3^. Despite advances in critical care, the mortality rate for sepsis and subsequent multiple organ dysfunction remains high, ranging from 25 to 30% in the last decade^4,5^. No effective pharmacological management is currently available.

The renin–angiotensin system is involved in homeostasis, with important roles in the modulation of blood pressure and hydro-electrolytes, and in various disease states^6,7^, including haemostasis^8,9^. High angiotensin (Ang) II expression induces pro-inflammatory cytokine and ROS production, apoptosis, endothelial dysfunction, and enhanced platelet activation and coagulation^10,11^. Ang-(1–7), an active peptide produced by Ang converting enzyme 2 (ACE2), counteracts various effects of Ang II via interaction with its G protein-coupled receptor Mas^6,12,13^. Thus, the ACE2/Ang-(1–7)/Mas axis can attenuate inflammation, ROS production, apoptosis, and organ dysfunction in pathological conditions^13,14^.

Increasing evidence both in vitro and in vivo indicates that Ang-(1–7) exerts anti-inflammatory effects through the Mas receptor^15–17^. In mouse peritoneal macrophages stimulated with endotoxin, Ang-(1–7) minimised the expression of tumour necrosis factor alpha and interleukin (IL)-6^15^. In various animal models, Ang-(1–7) protected against organ injury and mortality in polymicrobial sepsis^14^, acute lung injury^18^, and hypoxia-induced renal injury^16^.

Platelets appear to play an active role in the pathogenesis of multi-organ failure in sepsis^19^. A decline in platelet counts correlated with mortality in critically ill patients^20,21^. Furthermore, Ang-(1–7) exerts antithrombotic activity associated with Mas-mediated nitric oxide (NO) release from the endothelium and platelets^22,23^. However, little is known about the effects of Ang-(1–7) on platelet function in sepsis.

Lipopolysaccharide (LPS), a Gram-negative bacterial endotoxin, can induce the uncontrolled hyper-inflammatory response characteristic of sepsis, which frequently leads to multiple organ dysfunction^24^. In addition, endotoxaemia in rats can lead to significant thrombocytopaenia and increases in the thrombin–antithrombin complex, which is consistent with haemostatic disarrangement in septic patients^25,26^. Therefore, we used endotoxaemic rats to investigate whether Ang-(1–7) exerts protective effects against LPS-induced platelet dysfunction and organ injury. We also explored the potential mechanisms utilised by Ang-(1–7).

## Results

**Figure 1:**
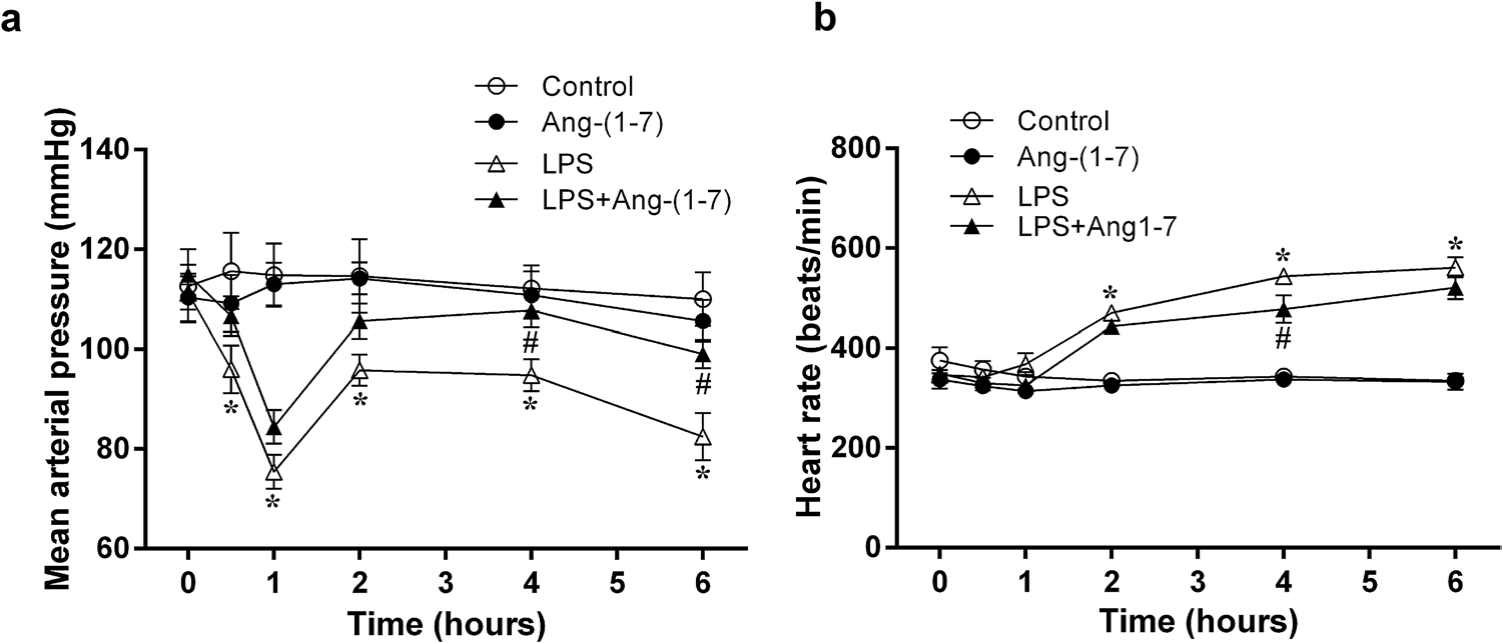
Effects of angiotensin-(1–7) on haemodynamic parameters. We assessed the changes in mean arterial blood pressure (**a**) and heart rate (**b**) during the experimental period in rats infused with 0.9% NaCl (control, n = 6), angiotensin (Ang)-(1–7) (1 mg/kg for 3 h, n = 6), *E. coli* lipopolysaccharide (LPS, 10 mg/kg for 15 min, n = 12), or LPS plus Ang-(1–7) (1 mg/kg for 3 h at 0.5 h after LPS, n = 12). The data are expressed as the mean ± standard error of the mean. *, p < 0.05 for LPS vs. control. ^#^, p < 0.05 for LPS + Ang-(1–7) vs. LPS.

**Figure 2:**
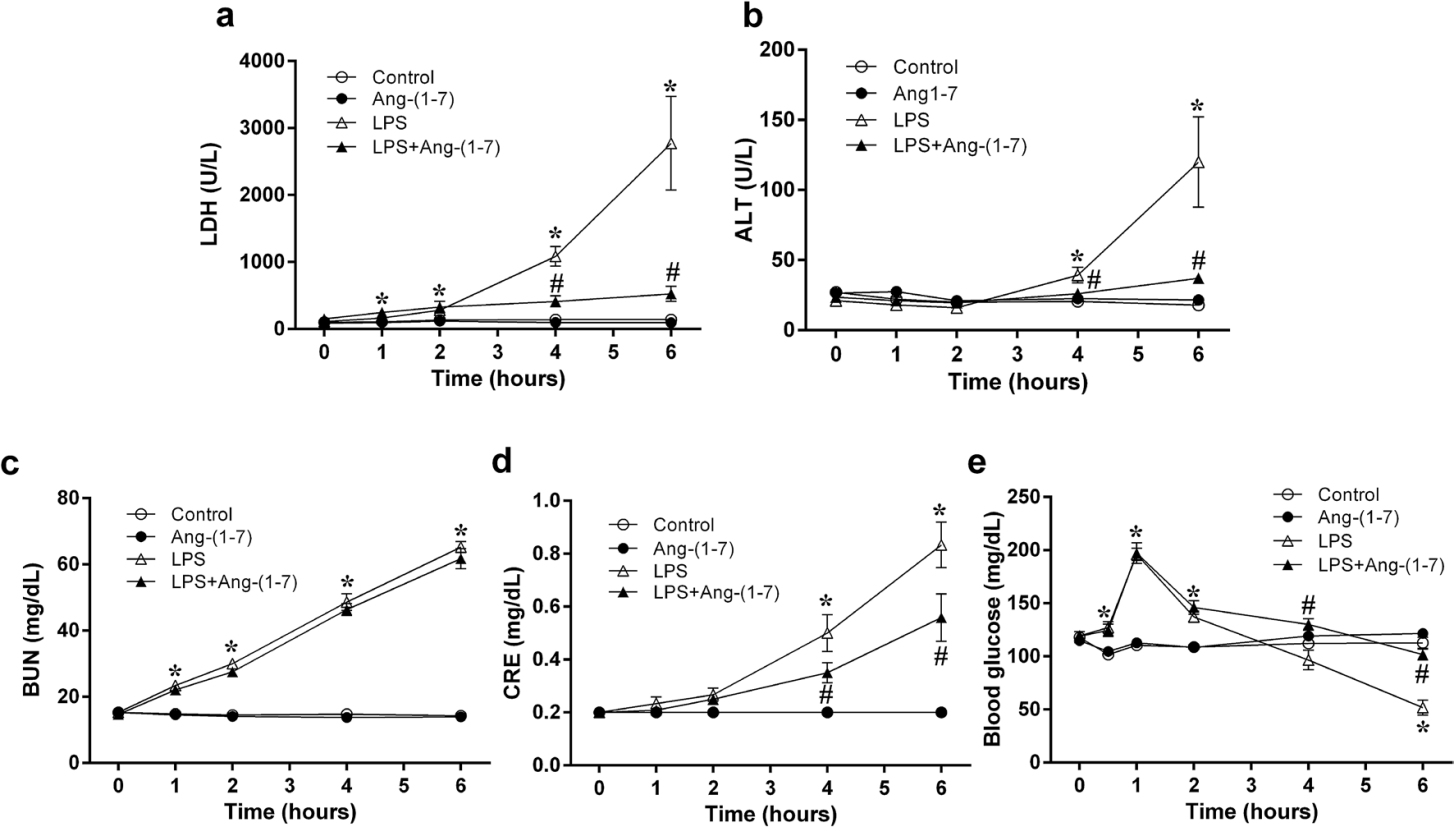
Angiotensin-(1–7) improves liver and kidney dysfunction and ameliorates delayed hypoglycaemia in endotoxaemic rats. We assessed the changes in lactate dehydrogenase (LDH) (**a**), alanine aminotransferase (ALT) (**b**), blood urea nitrogen (BUN) (**c**), creatinine (**d**), and blood glucose (**e**) levels during the experimental period in rats infused with 0.9% NaCl (control, n = 6), angiotensin (Ang)-(1–7) (1 mg/kg for 3 h, n = 6), *E. coli* lipopolysaccharide (LPS, 10 mg/kg for 15 min, n = 12), or LPS plus Ang-(1–7) (1 mg/kg for 3 h at 0.5 h after LPS, n = 12). The data are shown as the mean ± standard error of the mean. *, p < 0.05 for LPS vs. control. ^#^, p < 0.05 for LPS + Ang-(1–7) vs. LPS.

**Figure 3:**
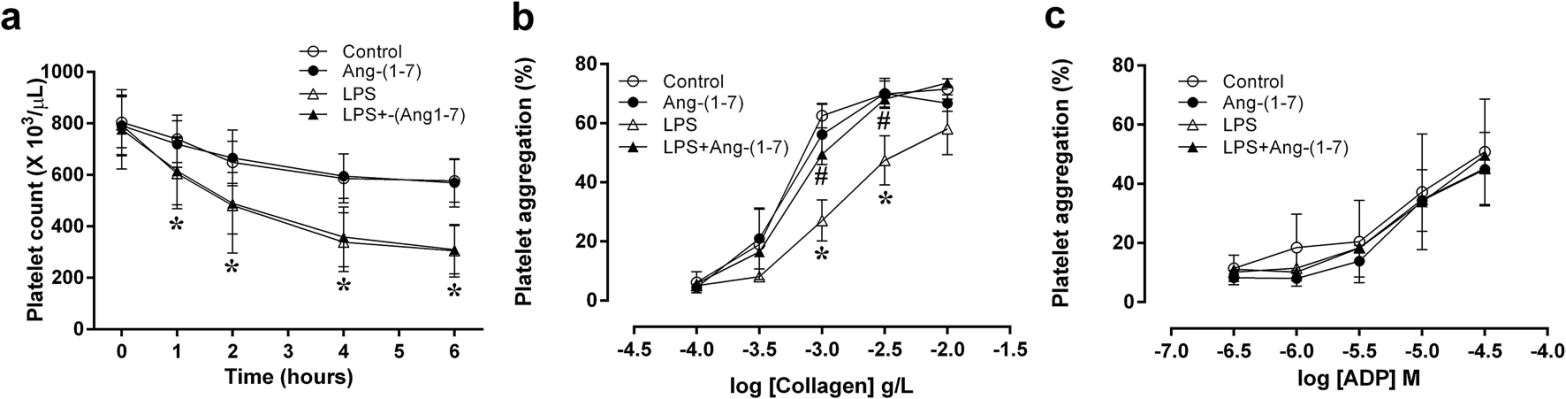
Effects of angiotensin-(1–7) on platelet counts and aggregation. We assessed the changes in platelet counts (**a**) during the experimental period in rats infused with 0.9% NaCl (control, n = 6), angiotensin (Ang)-(1–7) (1 mg/kg for 3 h, n = 6), *E. coli* lipopolysaccharide (LPS, 10 mg/kg for 15 min, n = 12), or LPS plus Ang-(1–7) (1 mg/kg for 3 h at 0.5 h after LPS, n = 12). Platelet aggregation was measured using various concentrations of collagen (**b**) and adenosine diphosphate (ADP) (**c**) to activate platelets from control (n = 4), Ang-(1–7) (n = 4), LPS (n = 9), or LPS + Ang-(1–7) (n = 9) rats at the end of the experiment (at 6 h). The data are shown as the mean ± standard error of the mean. *, p < 0.05 for LPS vs. control. ^#^, p < 0.05 for LPS + Ang-(1–7) vs. LPS.

**Figure 4:**
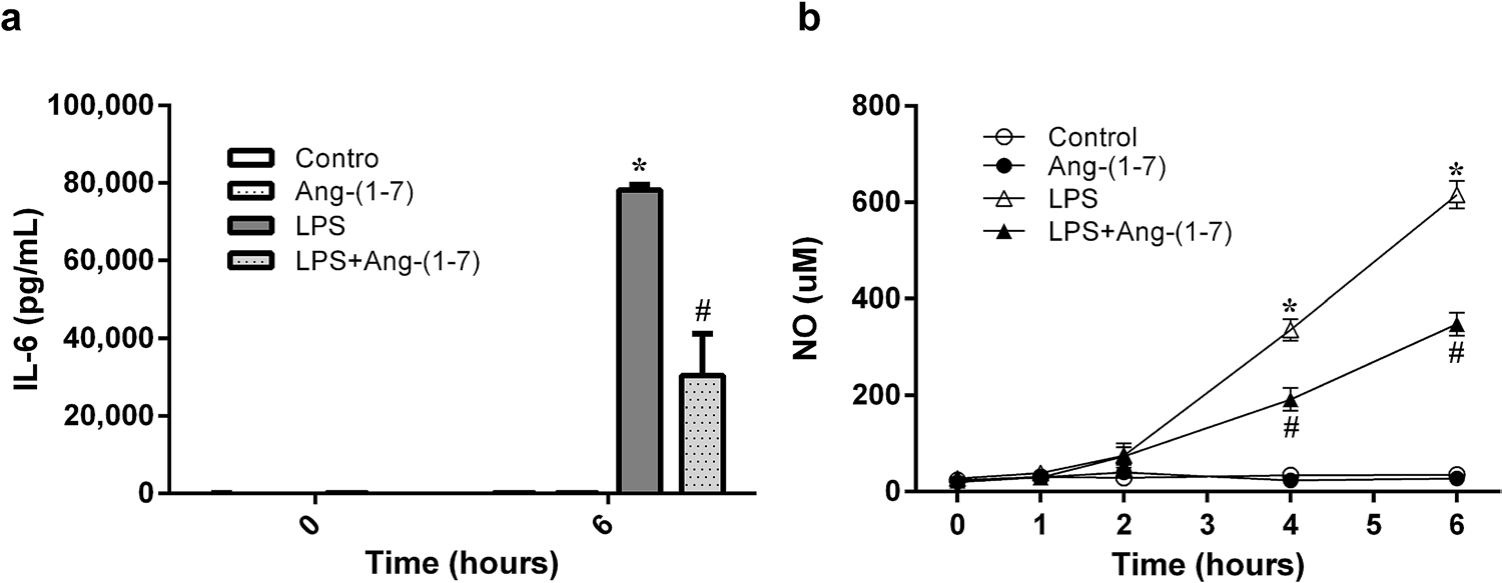
Angiotensin-(1–7) attenuates the elevated plasma levels of interleukin-6 and nitric oxide in endotoxaemic rats. We assessed the changes in interleukin (IL)-6 (**a**) in rats infused with 0.9% NaCl (control, n = 6), angiotensin (Ang)-(1–7) (1 mg/kg for 3 h, n = 6), *E. coli* lipopolysaccharide (LPS, 10 mg/kg for 15 min, n = 6), or LPS plus Ang-(1–7) (1 mg/kg for 3 h at 0.5 h after LPS, n = 6); or the changes in NO (**b**) during the experimental period in control (n = 6), Ang-(1–7) (n = 6), LPS (n = 12), or LPS + Ang-(1–7) (n = 12) rats. The data are shown as the mean ± standard error of the mean. *, p < 0.05 for LPS vs. control. ^#^, p < 0.05 for LPS + Ang-(1–7) vs. LPS.

We did not observe significant differences in the haemodynamic or biochemical parameters, platelet counts, or inflammatory markers among the groups at baseline, or between the control and Ang-(1–7) groups during the experimental period (Figs. 1, 2, 3, 4).

### Ang-(1–7) attenuated endotoxin-induced delayed hypotension in rats

Systemic LPS administration resulted in a biphasic fall in mean arterial pressure. The initial fall in arterial pressure appeared at 1 h, followed by a partial recovery, and then a further progressive fall from 4 to 6 h after LPS administration (Fig. 1a). Meanwhile, endotoxin caused a significant increase in heart rate from 2 to 6 h after administration (p < 0.001 vs. control group; Fig. 1b). Treatment with Ang-(1–7) significantly improved LPS-induced late hypotension (p = 0.009 at 4 h and p = 0.004 at 6 h vs. LPS group; Fig. 1a), but not tachycardia.

### Ang-(1–7) improved the plasma indexes of organ injury and blood glucose in endotoxaemic rats

At 4 and 6 h post-administration, LPS elevated plasma levels of lactate dehydrogenase (LDH), alanine aminotransferase (ALT), blood urea nitrogen (BUN), and creatinine (p < 0.05 vs. control group; Fig. 2a–d), indicating organ injury. Ang-(1–7) treatment significantly ameliorated these increased plasma parameters (p < 0.05 vs. LPS group; Fig. 2a–d).

After LPS administration, the levels of blood glucose initially increased at 1 h (p < 0.001) and subsequently declined below the baseline values (at 6 h: p < 0.001 vs. control group; Fig. 2e). Ang-(1–7) treatment attenuated LPS-induced late hypoglycaemia at 4 and 6 h after administration (at 4 h: p = 0.001 and at 6 h: p < 0.001 vs. LPS group; Fig. 2e), but not the initial hyperglycaemia.

### Ang-(1–7) improved platelet aggregation in endotoxaemic rats

LPS induced a significant decrease in platelet counts over time (p < 0.05 vs. control group; Fig. 3a), which was not ameliorated by Ang-(1–7) treatment (Fig. 3a). Furthermore, endotoxin significantly inhibited collagen-stimulated platelet aggregation (1 μg/ml collagen: p = 0.008 and 3 μg/ml collagen: p = 0.034 vs. control group; Fig. 3b). Importantly, treatment with Ang-(1–7) in endotoxaemic rats restored the LPS-induced decreases in platelet aggregation stimulated by collagen (1 μg/ml collagen: p = 0.029 and 3 μg/ml collagen: p = 0.014 vs. LPS group; Fig. 3b). In addition, treatment with Ang-(1–7) did not affect the platelet aggregation response curve activated by adenosine diphosphate (ADP) in the endotoxaemic rats compared to the control rats (Fig. 3c).

### Ang-(1–7) attenuated LPS-induced increases in plasma IL-6 and NO

**Figure 5:**
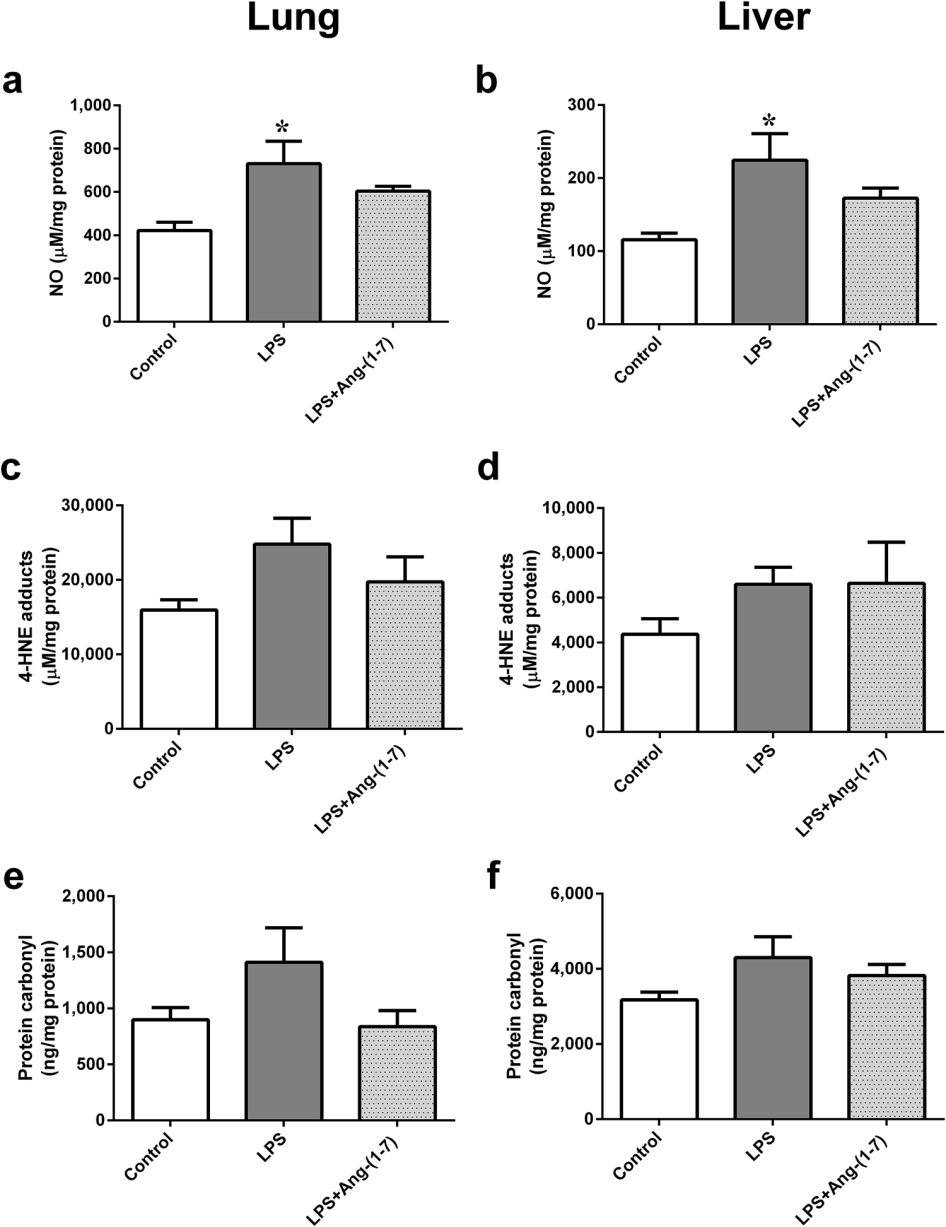
Effect of Angiotensin-(1–7) on tissue levels of nitric oxide (NO) and oxidative stress in endotoxaemic rats. We assessed the levels of NO (**a**,**b**), 4-hydroxynonenal (4-HNE) (**c**,**d**), and protein carbonyl (**e**,**f**) in lung and liver tissues in rats infused with 0.9% NaCl (control, n = 3), *E. coli* lipopolysaccharide (LPS, 10 mg/kg for 15 min, n = 3), or LPS plus Ang-(1–7) (1 mg/kg for 3 h at 0.5 h after LPS, n = 3). Lung and liver tissues were harvested 6 h after LPS administration. The data are shown as the mean ± standard error of the mean. *, p < 0.05 for LPS vs. control.

LPS significantly raised plasma IL-6 levels at 6 h post-administration (p < 0.001 vs. control group; Fig. 4a). This change was attenuated by Ang-(1–7) treatment (p = 0.001 vs. LPS group). In addition, LPS increased plasma NO levels with time (p < 0.001 vs. control group). The plasma NO increases at 4 and 6 h after LPS administration were also attenuated by Ang-(1–7) (at 4 h: p = 0.003 and at 6 h: p = 0.001 vs. LPS group; Fig. 4b). In lung and liver tissues, LPS also caused elevated NO levels in endotoxaemic rats (lung: p = 0.016 and liver: p = 0.016 vs. control group; Fig. 5a,b). Treatment with Ang-(1–7) attenuated the NO levels in tissues, and there was no significant difference of NO level between LPS + Ang-(1–7) and control group (Fig. 5a,b). As biomarkers of oxidative stress, the 4-hydroxynonenal (4-HNE) and protein carbonyl levels in lung and liver tissues were no significant differences among all groups (Fig. 5c–f).

### Ang-(1–7) minimized LPS-induced increases in CXCL1 and macrophage-inflammatory protein (MIP)-3α in lung tissues

**Figure 6:**
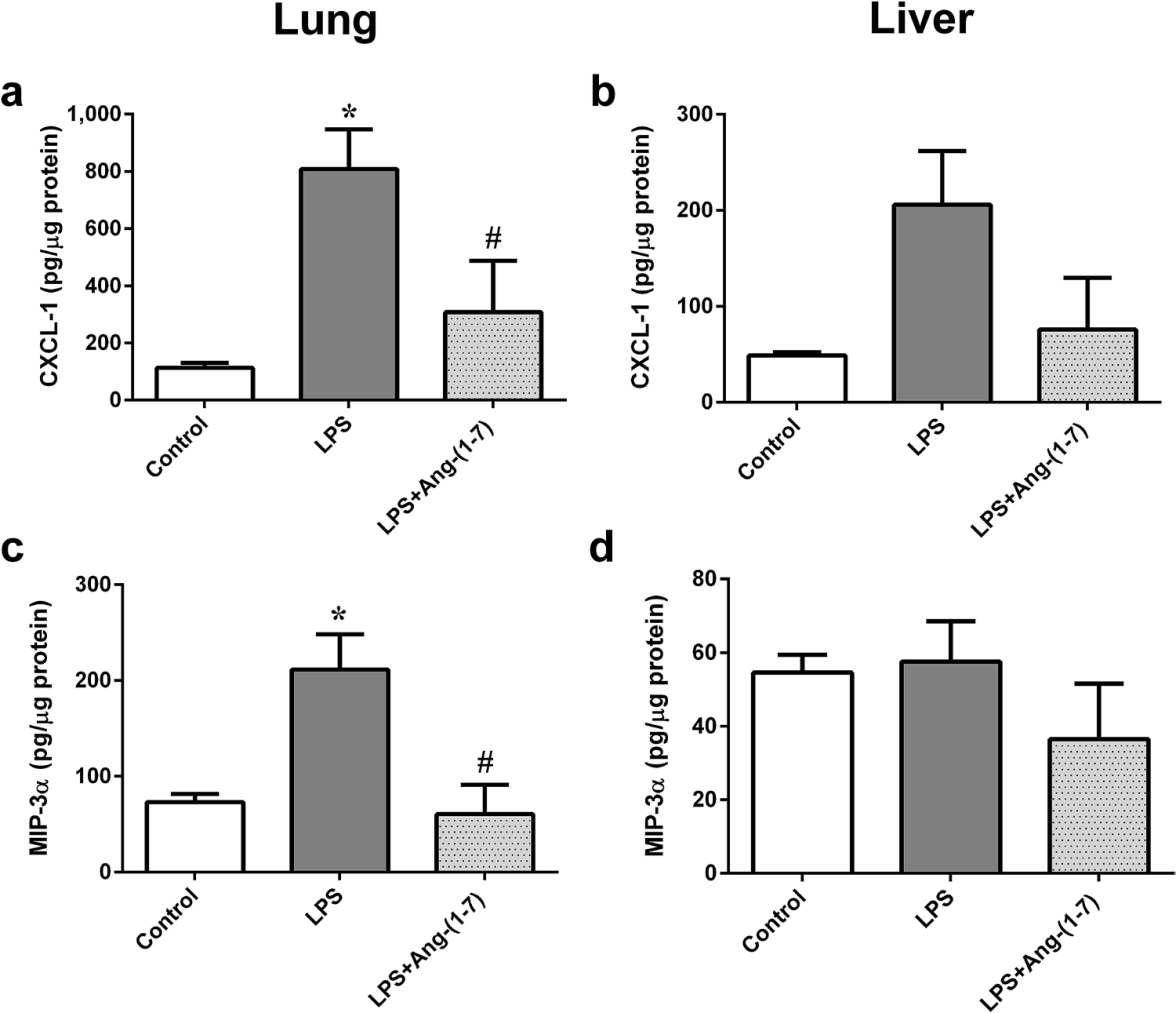
Angiotensin-(1–7) attenuated elevated tissue levels of CXCL1 and macrophage-inflammatory protein (MIP)-3α in endotoxaemic rats. We assessed the changes in lung CXCL1 (**a**), liver CXCL1 (**b**), lung MIP-3α (**c**), and liver MIP-3α levels in rats infused with 0.9% NaCl (control, n = 3), *E. coli* lipopolysaccharide (LPS, 10 mg/kg for 15 min, n = 3), or LPS plus Ang-(1–7) (1 mg/kg for 3 h at 0.5 h after LPS, n = 3). Lung and liver tissues were harvested 6 h after LPS administration. The data are shown as the mean ± standard error of the mean. *, p < 0.05 for LPS vs. control. ^#^, p < 0.05 for LPS + Ang-(1–7) vs. LPS.

Infiltrating neutrophils are directed to and activated in injured tissues by chemokines. CXCL1 and MIP-3α are two chemokines modulated by nuclear factor-kappa B (NF-κB)^27^. We observed significant concentration increases of CXCL1 and MIP-3α in lungs of LPS rats (CXCL1, p = 0.01 and MIP-3α, p = 0.013 vs. control group; Fig. 5a,c), which was attenuated by Ang-(1–7) (CXCL1, p = 0.036 and MIP-3α, p = 0.009 vs. LPS group; Fig. 6a,c). No significant differences of CXCL1 and MIP-3α in livers were noted among all groups (Fig. 6b,d).

### Ang-(1–7) attenuated LPS-induced changes in inducible nitric oxide synthase (iNOS) and IκB expression in endotoxaemic rats

**Figure 7:**
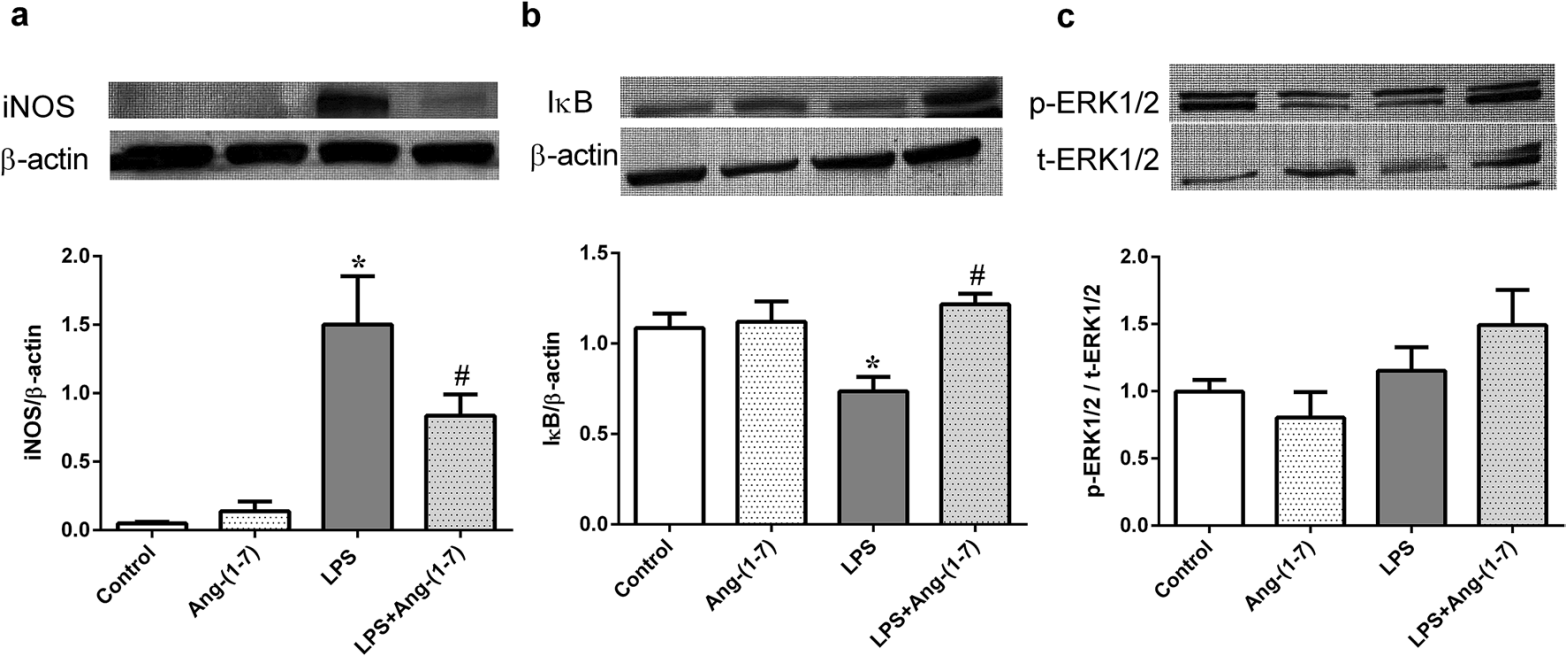
Ang-(1–7) attenuates the increased expression of inducible nitric oxide synthase and IκB, but not extracellular signal-regulated kinase 1/2, in endotoxaemic rats. We assessed the changes in inducible nitric oxide synthase (iNOS) (**a**), cytoplasmic IκB (**b**), and extracellular signal-regulated kinase (ERK) 1/2 (**c**) in the liver tissues of rats infused with 0.9% NaCl (control), angiotensin (Ang)-(1–7) (1 mg/kg for 3 h), *E. coli* lipopolysaccharide (LPS, 10 mg/kg for 15 min), or LPS plus Ang-(1–7) (1 mg/kg for 3 h at 0.5 h after LPS). Liver tissues were harvested 24 h after LPS administration. Representative blots are shown in the upper panel of the figure. β-actin and total ERK1/2 served as loading controls for iNOS and IκB and for phosphorylated ERK1/2, respectively. The data are shown as the mean ± standard error of the mean from 4 rats per group. *, p < 0.05 for LPS vs. control. ^#^, p < 0.05 for LPS + Ang-(1–7) vs. LPS.

LPS significantly increased iNOS expression and decreased IκB expression in liver tissues (p < 0.001 and p = 0.013, respectively, vs. control group; Fig. 7a,b). Ang-(1–7) treatment attenuated these changes in protein expression (iNOS: p = 0.033 and IκB: p = 0.002 vs. LPS group, respectively; Fig. 7a,b). However, neither phosphorylated extracellular signal-regulated kinase (ERK) 1/2 expression in livers (Fig. 7c) nor phosphorylated-p38 mitogen-activated protein kinase (MAPK) expression in lungs and livers (Supplemental Fig. 1) showed obvious differences among all groups.

### Histological examination/immunohistochemistry analysis

**Figure 8:**
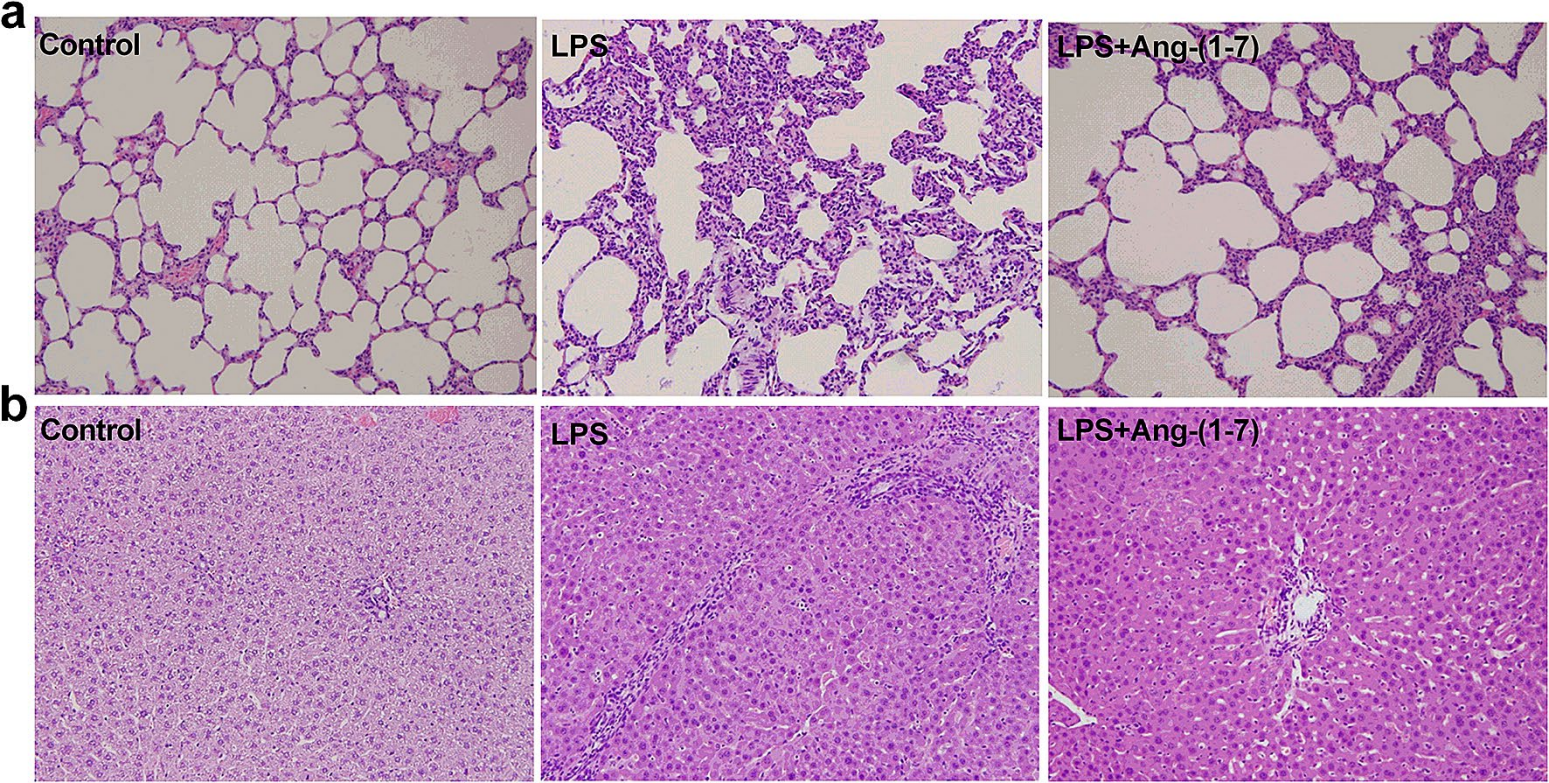
Histological analysis of lung and liver. Haematoxylin and eosin staining on (**a**) lung and (**b**) liver sections from rats infused with 0.9% NaCl (control), *E. coli* lipopolysaccharide (LPS, 10 mg/kg for 15 min), or LPS plus Ang-(1–7) (1 mg/kg for 3 h at 0.5 h after LPS, LPS + Ang-(1–7)). Shown are representative micrographs from 3 independent experiments in which the same results were obtained. Magnification × 200.

**Figure 9:**
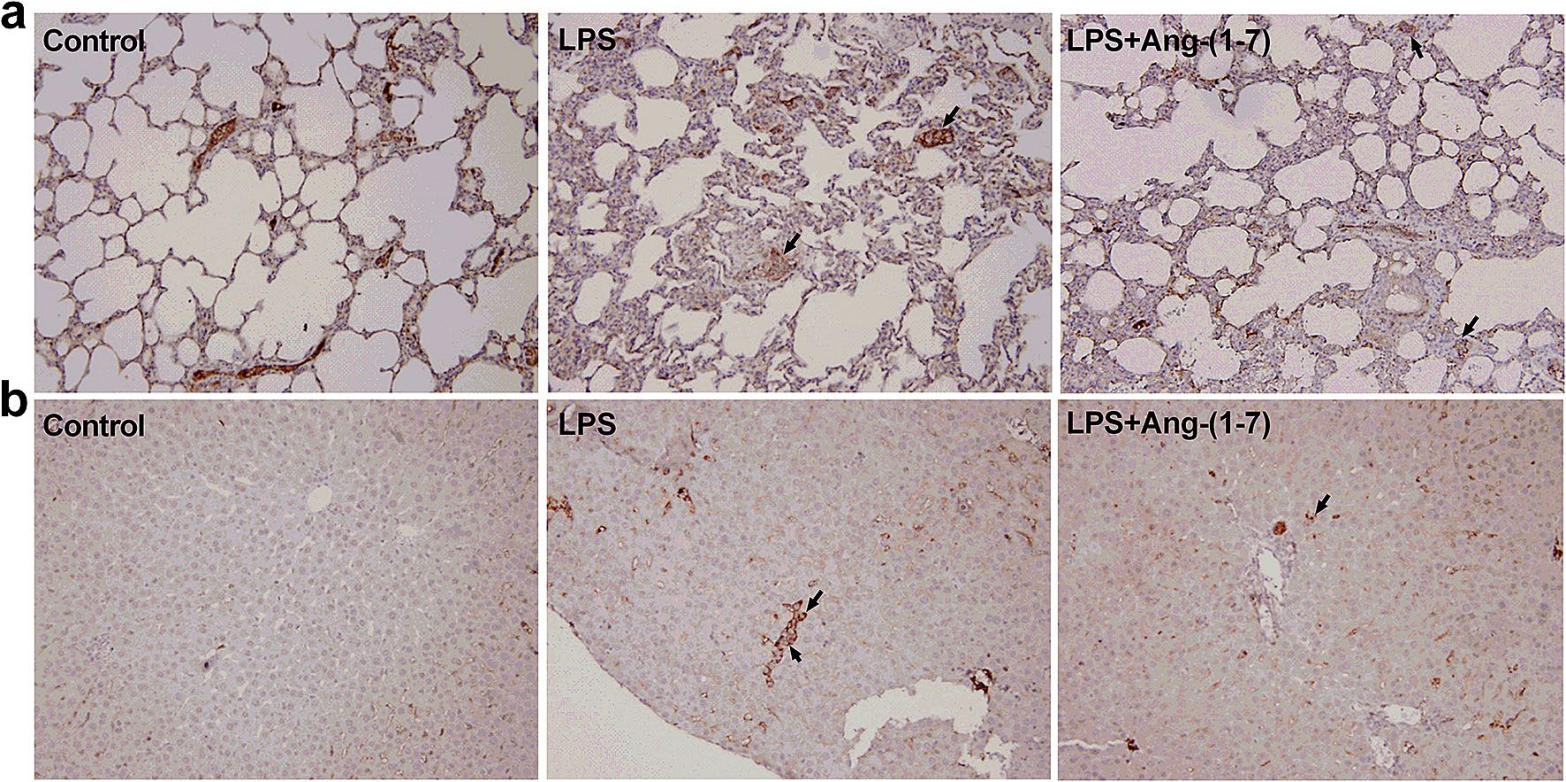
Effect of Ang-(1–7) on NF-κB p65 activation in lung and liver tissues in endotoxaemic rats. Immunohistochemistry staining for phosphorylated- NF-κB p65 in lung (**a**) and liver (**b**) tissues in rats infused with 0.9% NaCl (control), *E. coli* lipopolysaccharide (LPS, 10 mg/kg for 15 min), or LPS plus Ang-(1–7) (1 mg/kg for 3 h at 0.5 h after LPS, LPS + Ang-(1–7)). Arrows indicate positive staining. Samples were obtained at 6 h after LPS. Shown are representative photographs from 3 independent experiments in which the same results were obtained. Magnification × 200.

In histologic examination, LPS induced extensive alveolar interstitial fibrosis in lungs (Fig. 8a) and portal fibrosis with inflammation in liver tissues (Fig. 8b). However, treatment with Ang-(1–7) attenuated these histopathological changes. Immunohistochemistry (IHC) analysis was carried out to detect phosphorylated NF-κB expression in lung and liver tissue. We found that phosphorylated NF-κB was not expressed in lung (Fig. 9a) and liver (Fig. 9b) tissues in the control group. Moreover, the areas of phosphorylated NF-κB expression were increased in lung and liver tissues after LPS, and treatment with Ang-(1–7) in LPS rats decreased these areas (Fig. 9).

## Discussion

**Figure 10:**
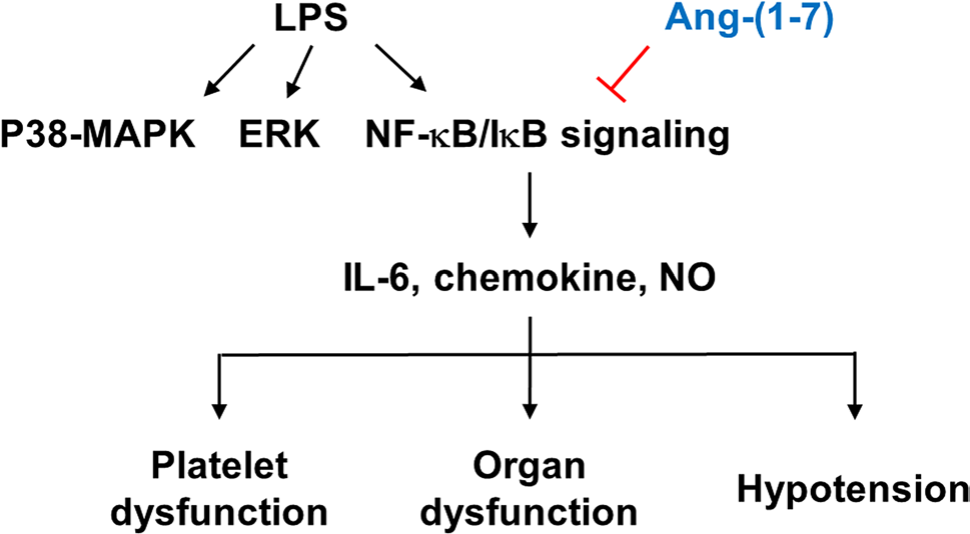
Schematic diagram of the effects of angiotensin-(1–7) in rats with endotoxaemia. ERK: extracellular signal-regulated kinase; IL: interleukin; NF-κB; nuclear factor kappa B; NO: nitric oxide; p38 MAPK: p38 mitogen-activated protein kinase.

We found that Ang-(1–7) administration inhibited endotoxin-induced hypotension, hypoglycaemia, thrombocytopathy, and multiple organ injury in rats. Along with the findings that Ang-(1–7) altered inflammatory and signalling molecule expression, these results suggest that Ang-(1–7) ameliorates LPS-induced inflammation and platelet dysfunction, thereby protecting against organ injury. This hypothesis and the results of the study are summarised in Fig. 10.

The underlying mechanism through which Ang-(1–7) mediates its protective effects against sepsis remains unresolved. In the classical pathway, NF-κB proteins are bound and inhibited by IκB proteins. Upon stimulation by LPS, IκB is phosphorylated and degraded, and thus allowing NF-kB activation. Active NF-κB complexes are further activated by phosphorylation, and translocate to the nucleus, where it regulates the target gene expression. The ACE2/Ang-(1–7)/Mas axis protects pancreatic acinar cells from inflammatory damage by inhibiting the p38 MAPK/NF-κB pathway^28^, but previous studies showed that Ang-(1–7) counteracts LPS-induced skeletal muscle atrophy^29^ and hepatocyte injury^30^ partially by signalling through the p38 MAPK pathway. Furthermore, Ang-(1–7) inhibits ERK1/2 and NF-κB activation to protect against LPS-induced acute lung injury in rats^31^. However, we found that Ang-(1–7) increased IκB protein and suppressed phosphorylated NF-κB expression, but not activation of the ERK1/2 and p38 pathway in livers and lungs of endotoxaemic rats. Our findings suggest that the anti-inflammatory mechanism of Ang-(1–7) may differ by cell type and in physiological versus pathological conditions^32^.

Among three isoforms of NOS, nNOS and eNOS produce a nanomolar amount of NO for seconds to minutes, while iNOS generate large amounts of NO (micromolar range) by stimulating with proinflammatory cytokines and LPS, sometimes for hours^33–35^. Indeed, we found LPS administration increased the plasma NO levels and significantly increased iNOS expression in liver tissues, which were attenuated by Ang-(1–7). NF-κB signalling modulates the transcription of inflammatory genes, including pro-inflammatory cytokines, chemokines and iNOS^36,37^. Thus, Ang-(1–7) may act through the IκB/NF-κB pathway to attenuate IL-6, chemokines and iNOS production in the setting of sepsis.

Sepsis induces complex regulation of platelet functions, such as alterations in aggregation^38^. Incubation of human platelets with LPS for 1 h inhibited aggregation by suppressing Ca^2+^ mobilisation and protein kinase C activation^39^, whereas 6-h incubation of rat platelets with LPS did not induce activation and aggregation^40^. We found that platelet aggregation induced by collagen, but not by ADP, was suppressed in endotoxaemic rats at 6 h post-administration, which is comparable to the findings of human sepsis studies^38,41^. Two mechanisms may account for this finding: (1) only hyporesponsive platelets remained at the end stage of endotoxaemia in rats, or (2) platelets were desensitised to various agonists during the course of sepsis^42,43^. Further studies are necessary to clarify the possible mechanisms that modulate platelet responses to various agonists in sepsis.

Activated platelets release thrombin, leading to platelet aggregation and three-dimensional thrombus formation, which can be inhibited by ACE inhibitors and angiotensin type I receptor blockers through Ang-(1–7) production^44^. Furthermore, Ang-(1–7) exerts antithrombotic activity by signalling through the Mas receptor to stimulate NO release from platelets and endothelial cells^22^. Interestingly, platelets do not have the nuclear transcriptional regulatory mechanisms of the iNOS gene, however, iNOS knock-out and iNOS inhibitors modulate collagen-induced platelet aggregation in vitro^45^. In addition, platelets are endowed with the NF-κB/I-κBα complex. NF-κB regulates platelet phospholipase C-β2^46^, and inhibitors of NF-κB modulate platelet activation^47^. Ang-(1–7) administration ameliorated the endotoxin-induced inhibition of platelet aggregation in our study, while dampening iNOS expression and consequent NO overproduction. These findings indicate that Ang-(1–7) affects platelet aggregation in endotoxaemic rats through its global effects rather than through its antithrombotic activity.

Although Ang-(1–7) administration improved LPS-induced suppression of collagen-activated platelet aggregation, it did not attenuate LPS-induced thrombocytopaenia. Altered platelet production and scavenging in the circulation during sepsis and increased platelet consumption by DIC have all been attributed to thrombocytopaenia^40^. These complicated mechanisms of thrombocytopaenia action may explain why Ang-(1–7) did not improve endotoxin-induced thrombocytopaenia.

In a preliminary study, we found that 2 mg/kg of Ang-(1–7) resulted in haemodynamic instability and even early death in endotoxaemic rats (data not shown). Thus, we used 1 mg/kg of Ang-(1–7) infused over 3 h, a dosage comparable to our previous study in a caecal ligation/perforation (CLP) model^14^. This dosage did not exert marked effects on haemodynamic or biochemical variables in control rats during the experimental period.

CLP-induced polymicrobial sepsis and endotoxaemia promote microvascular thrombosis through different mechanisms^26^. Kinasewitz et al. reported that endotoxin rather than CLP leads to thrombocytopenia and coagulation derangement, which were comparable to the haemostasis characteristics of human sepsis^25^. Thus, we chose the animal model of endotoxaemia, which induces prolonged thrombocytopaenia, platelet sequestration, and pro-inflammatory cytokine responses, to assess the effects of Ang-(1–7) on platelet function, inflammatory responses, and the related mechanism in sepsis^41^. In addition, we used only male rats in this study to avoid the confounding effects of estrous cycle and sex hormones in female rats. Further studies using female animals are warranted to investigate the role of Ang-(1–7) in sepsis.

In conclusion, Ang-(1–7) treatment inhibited multiple organ injury in endotoxaemic rats by attenuating inflammation, diminishing NO production, and restoring LPS-induced platelet dysfunction. The IκB/NF-κB signalling pathway, rather than the ERK1/2 and p38 pathway, appears to mediate the protective effects of Ang-(1–7) in this model. Our observations suggest that the Ang-(1–7)/Mas axis should be considered a putative target for the development of a new class of drugs to treat sepsis. Additional studies are needed to explore the molecular mechanism of the Ang-(1–7)/Mas pathway in platelets during sepsis, such as studies using *Mas*^−/−^ platelets in *Mas*^+/+^ mice.

## Materials and methods

### Animals and experimental design

Thirty-six adult male Wistar rats (260 to 280 g; BioLASCO Taiwan, Taipei, Taiwan) were used in this study. The rats were housed in 12-h light/dark conditions with free access to food and tap water. The animal studies complied with the National Institutes of Health guidelines for ethical animal treatment, and were approved by the Committee on the Ethics of Animal Experiments of Taipei Veterans General Hospital (IACUC-2015-164).

We anesthetised the rats by intraperitoneal injection of sodium pentobarbital (50 mg/kg). We placed a polyethylene catheter in the left carotid artery, for arterial pressure monitoring, and in the right jugular vein, for drug administration. The catheters were cannulated, exteriorised, and fixed to the back of the neck. The cannulated animals were allowed to recover to their normal condition overnight with ad libitum access to standardised pellet food and tap water.

After we recorded baseline haemodynamics and collected blood, we randomly divided the rats into 4 groups. The control group received intravenous infusion of normal saline (1 ml/kg) for 15 min from 0 h, the Ang-(1–7) group received intravenous infusion of Ang-(1–7) (1 mg/kg for 3 h) starting 30 min after saline infusion, the LPS group received intravenous infusion of *Escherichia coli*-derived LPS (10 mg/kg for 15 min) starting from 0 h, and the LPS + Ang-(1–7) group received infusion of Ang-(1–7) (1 mg/kg for 3 h) starting 30 min after the same LPS regimen. The dosage of Ang-(1–7) was based on our previous study^14^.

Bacterial LPS (*E. coli* serotype 0127:B8; L3127) was purchased from Sigma Chemical (St Louis, MO, USA) and Ang-(1–7) was obtained from Tocris Bioscience (Bristol, UK). The experiments were performed on pairs of rats, and the haemodynamic parameters were monitored for 6 h. At the 0, 1, 2, 4 and 6 h-time points, we obtained 0.8 ml of blood from experimental rats for biochemistry, cytokines, and platelet counts studies. Each volume of blood withdrawn was replaced with an equal volume of saline. We assessed signs of distress in the animals hourly during the experimental period. At 6 h after LPS treatment or upon signs of imminent death (i.e. failure to respond to external stimuli, inability to maintain upright position/tremors, prolonged/deep hypothermia, and/or agonal breathing), rats were sacrificed by overdose of pentobarbital (100 mg/kg, intravenous). We excised the lungs and livers to analyse the levels of iNOS, IκB, and ERK1/2 protein expression.

### Measurement of haemodynamic parameters

We monitored haemodynamics in all rats by connecting the arterial catheter to a pressure transducer (P23ID, Statham, Oxnard, CA, USA). At 0, 0.5, 1, 2, 4, and 6 h after LPS treatment, we recorded phasic blood pressure and heart rate on a multichannel recorder (XctionView Data Acquisition System, SINGA Technology Corporation, Taipei, Taiwan).

### Quantification of organ function and injury

We analysed the blood glucose levels in the samples (10 μl) using the OneTouch II blood glucose monitoring system (Lifescan, Milpitas, CA, USA). After immediate centrifugation at 16,000 g for 3 min, some samples were analysed for plasma levels of LDH, ALT, BUN, and creatinine using a Fuji DRICHEM 3030 (Fuji Photo Film, Tokyo, Japan). Each volume of blood withdrawn was replaced with an equal volume of saline.

### Measurement of platelet counts

Whole blood samples were collected 10:1 (v/v) in acid citrate dextrose solution (1.5 mM citric acid, 8.5 mM sodium citrate, and 13.6 mM dextrose). We measured platelet counts with an automated haematology analyser (Sysmex KX-21 N Sysmex America, Mundelein, IL, USA).

### Washed platelet preparation

It was essential to examine platelet aggregation using washed platelets to isolate the cells from the effects of the plasma environment^48^. We collected and centrifuged (100 g for 15 min) whole blood samples to obtain platelet-rich plasma (PRP) at the end of the experiment (6 h post-LPS administration). Platelets were pelleted by further centrifugation of the PRP in the presence of apyrase (0.02 U/ml) and prostaglandin I_2_ (1 μg/ml) at 700 g for 10 min. The resulting pellets were resuspended twice in modified Tyrode’s buffer (134 mM NaCl, 2.9 mM KCl, 12 mM NaHCO_3_, 0.34 mM NaH_2_PO_4_, 1 mM MgCl_2_, 20 mM HEPES, 0.1% glucose, and 0.35% bovine serum albumin). The washed platelets were adjusted to a concentration of 3 × 10^5^ cells/μL.

### Platelet aggregometry

Platelet aggregation was monitored in 96-well plates using a modified light transmission method^49^. Briefly, we placed washed platelets (100 μl aliquots) into individual wells of 96-well plates. We added various concentrations of ADP (0.3–30 μM) and collagen (0.1–10 μg/ml) to the wells, which we shook on a microplate shaker (BioShake IQ, Quantifoil Instruments, Großlöbichau, Germany) at 1200 rpm for 5 min at 37 °C to activate the platelets. We measured the absorbance at 595 nm with a 96-well plate reader (Tecan Sunrise; Tecan, Weymouth, UK). We calculated the percentage of platelet aggregation using the absorbance of modified Tyrode’s buffer as a blank.

### Measurement of plasma IL-6 concentrations

Rat plasma samples obtained at 0 and 6 h of treatment were used to measure IL-6 levels with an enzyme-linked immunosorbent assay kit (R&D Systems, Minneapolis, MN, USA) according to the manufacturer’s instructions.

### Tissue homogenization and protein quantification

The lungs and livers obtained after euthanasia were homogenized in lysis buffer and centrifuged for 25 min at 18,000 × *g* (4 °C). Protein in the supernatant portion was quantitated by BCA Protein Assay Kit (Thermo scientific, Rockford, IL, USA). We used aliquots of tissue homogenates for tissue chemokine levels, NO assay, oxidative stress and Western blot analysis.

### Measurement of chemokine levels in tissue

The CXCL1 and MIP-3α levels in lung and liver tissues were determined using specific ELISA kits from R&D Systems (Abingdon, UK) according to manufacturer’s instructions.

### Measurement of NO concentrations

We measured total nitrite and nitrate concentrations in plasma and tissue homogenates to determine NO concentration. We incubated plasma (30 μl) and tissue homogenates with 95% ethanol for 30 min, and then centrifuged the samples at 16,000 g for 6 min. The nitrite/nitrate concentrations in all samples were analysed by chemiluminescence as described in previous studies^50,51^. Briefly, to measure the amounts of nitrate, the supernatant portion was added to a reducing agent (0.8% VCL_3_ in 1 N HCl) in the purge vessel to convert nitrate to NO·. NO· was stripped from the samples using helium purge gas, and was then drawn into an NO analyser (Sievers NOA-280i; Sievers, Boulder, CO, USA). Nitrate concentrations in plasma and tissues were calculated after comparison with standard solutions of sodium nitrate (Sigma Chemical, St Louis, MO, USA).

### Oxidative stress analysis

The 4-HNE adducts and protein carbonyl levels in lung and liver tissues were measured using ELISA kits from Mybiosource (San Diego, CA, USA) according to manufacturer’s instructions.

### Western blot analysis

To determine the protein expression of iNOS, IκB, and ERK1/2, we obtained liver tissues after euthanasia. Homogenised lung and liver tissues supernatants (100 μg total protein) were subjected to 10–12% polyacrylamide gel electrophoresis and transferred onto nitrocellulose membranes (Mini Trans-Blot Cell, Bio-Rad Laboratories, Hercules, CA, USA). After blocking with 5% albumin (BioShop Canada, Burlington, ON, Canada) in Tris-buffered saline containing 0.1% TWEEN 20 (TBST) for 1.5 h at room temperature, we incubated the membranes with a primary antibody against rat iNOS (1:1,000; BD Transduction Laboratories, Lexington, KY, USA), IκB (1:3,000; Abcam, Cambridge, UK), ERK1/2 (1:3,000; Genetex, Irvine, CA, USA), phosphorylated ERK1/2 (1:1,000 dilution, Genetex, Irvine, CA, USA), or phosphorylated p38 MAPK (1:4,000; Arigo) in TBST buffer at 4 °C overnight. The blots were then incubated in appropriate horseradish peroxidase-conjugated secondary antibodies (Cell Signaling Technology, Danvers, MA, USA). The protein expression was detected using an enhanced chemiluminescence western blotting reagent (Thermo Scientific, Rockford, IL, USA), and imaged with radiographic film. We quantified the protein in the bands by densitometry. In order to save the reagent and antibody, we used adequate length of membrane instead of full-length membrane for albumin blocking and antibody incubation. Therefore, we cropped the adequate length of membrane according to the marker which meets the molecular weight of targeted protein after transferring onto nitrocellulose membrane.

### Histological examination/IHC analysis

Lung and liver specimens were fixed in 10% formaldehyde for more than 24 h, which was followed by dehydration in graded ethanol. The tissues were embedded in paraffin wax, sectioned into 4 µm-thick slices and stained with haematoxylin and eosin. For IHC analysis, the sections were blocked with goat serum and then incubated with the anti-p-NF-κB p65 (Ser536) (1:200, Cell Signaling Technology, Inc., Danvers, MA, USA). The color reaction was developed using DAB solution. Finally, the histologic alterations and IHC samples were evaluated by a pathologist in a blinded fashion.

### Statistical analysis

We used Shapiro–Wilk tests to test variable distribution, and we conducted logarithmic transformation for variables that did not follow the normal distribution. Comparisons of physiological parameters among the groups were performed using the analysis of variance for repeated measures. We compared differences in the protein expression of iNOS, IκB, and ERK1/2 with the one-way analysis of variance combined with the least significant difference post hoc test. The data are presented as the mean ± standard error of the mean. A *p* value < 0.05 was considered statistically significant.

## Supplementary Information

Supplementary Information

## Data Availability

All data generated or analysed in this study are included in this published article.
