# Supplementary material for: Angiotensin-(1–7) treatment blocks lipopolysaccharide-induced organ damage, platelet dysfunction, and IL-6 and nitric oxide production in rats
Source: Sci Rep. 2021 Jan 12;11:610. doi: 10.1038/s41598-020-79902-x (PMC7804205; doi:10.1038/s41598-020-79902-x)
Supplement: Supplementary file 1 — Supplementary Information [file 41598_2020_79902_MOESM1_ESM.pptx]

## Slide 1
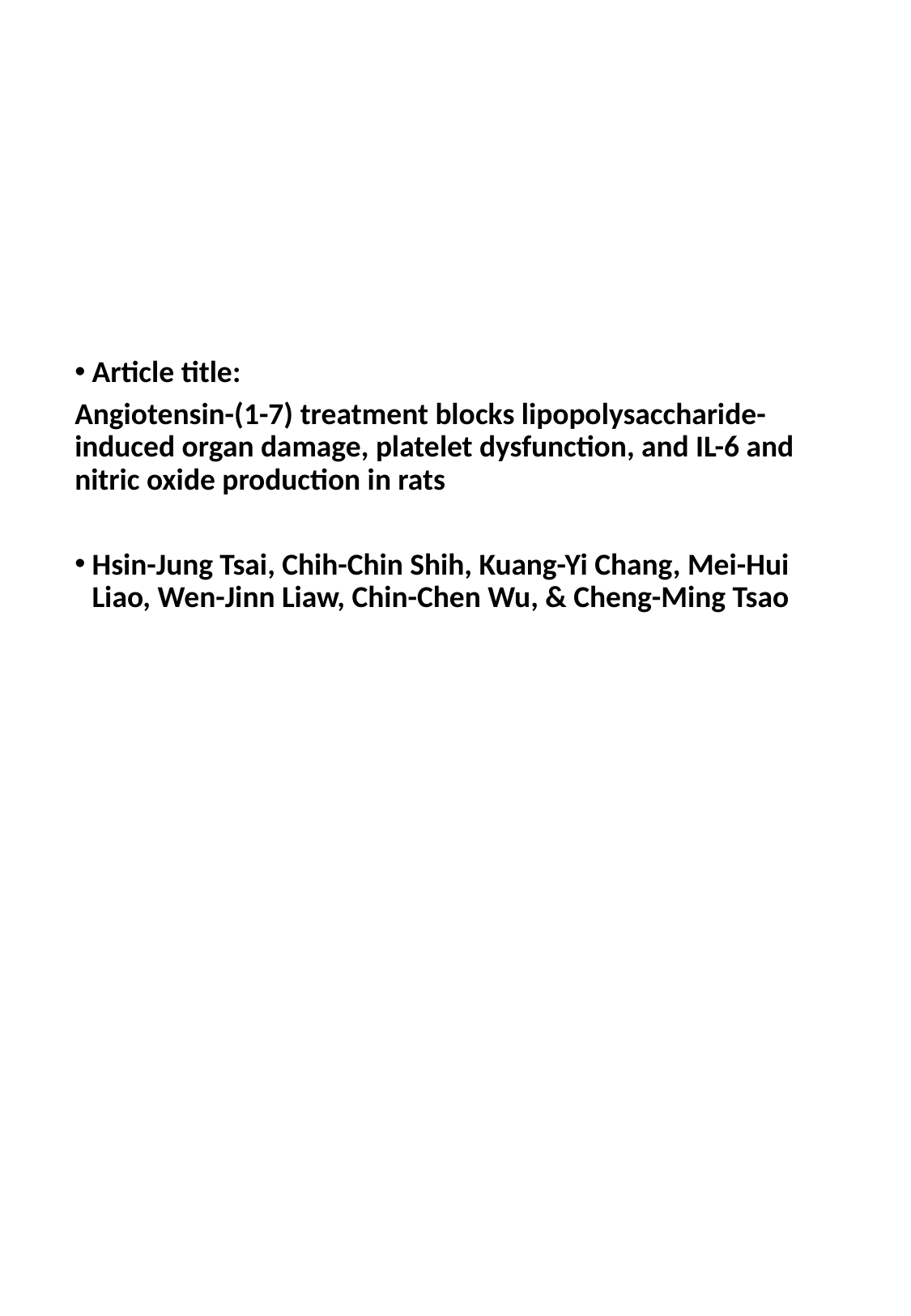

Article title:
Angiotensin-(1-7) treatment blocks lipopolysaccharide-induced organ damage, platelet dysfunction, and IL-6 and nitric oxide production in rats
Hsin-Jung Tsai, Chih-Chin Shih, Kuang-Yi Chang, Mei-Hui Liao, Wen-Jinn Liaw, Chin-Chen Wu, & Cheng-Ming Tsao

## Slide 2
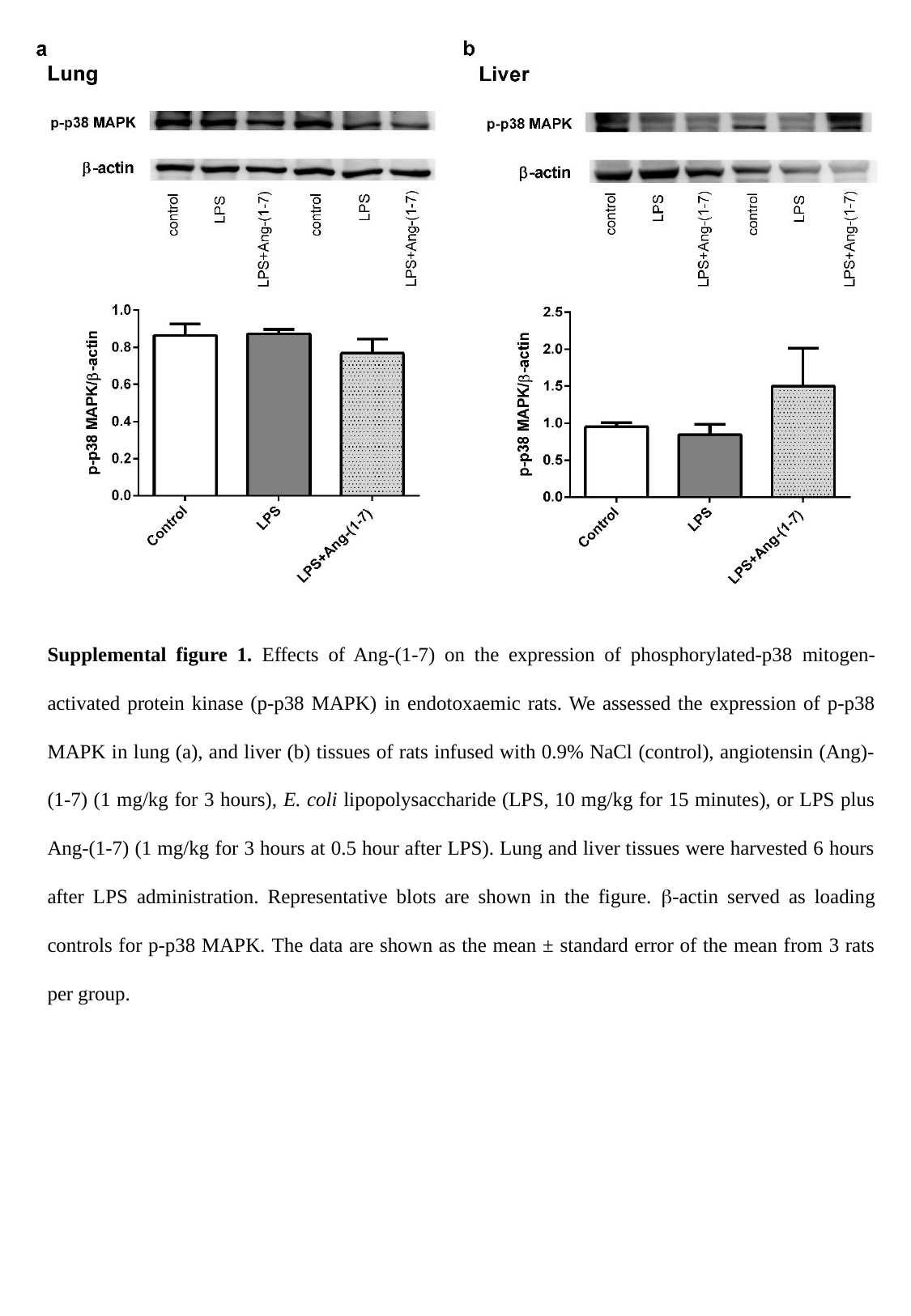

Supplemental figure 1. Effects of Ang-(1-7) on the expression of phosphorylated-p38 mitogen-activated protein kinase (p-p38 MAPK) in endotoxaemic rats. We assessed the expression of p-p38 MAPK in lung (a), and liver (b) tissues of rats infused with 0.9% NaCl (control), angiotensin (Ang)-(1-7) (1 mg/kg for 3 hours), E. coli lipopolysaccharide (LPS, 10 mg/kg for 15 minutes), or LPS plus Ang-(1-7) (1 mg/kg for 3 hours at 0.5 hour after LPS). Lung and liver tissues were harvested 6 hours after LPS administration. Representative blots are shown in the figure. b-actin served as loading controls for p-p38 MAPK. The data are shown as the mean ± standard error of the mean from 3 rats per group.

## Slide 3
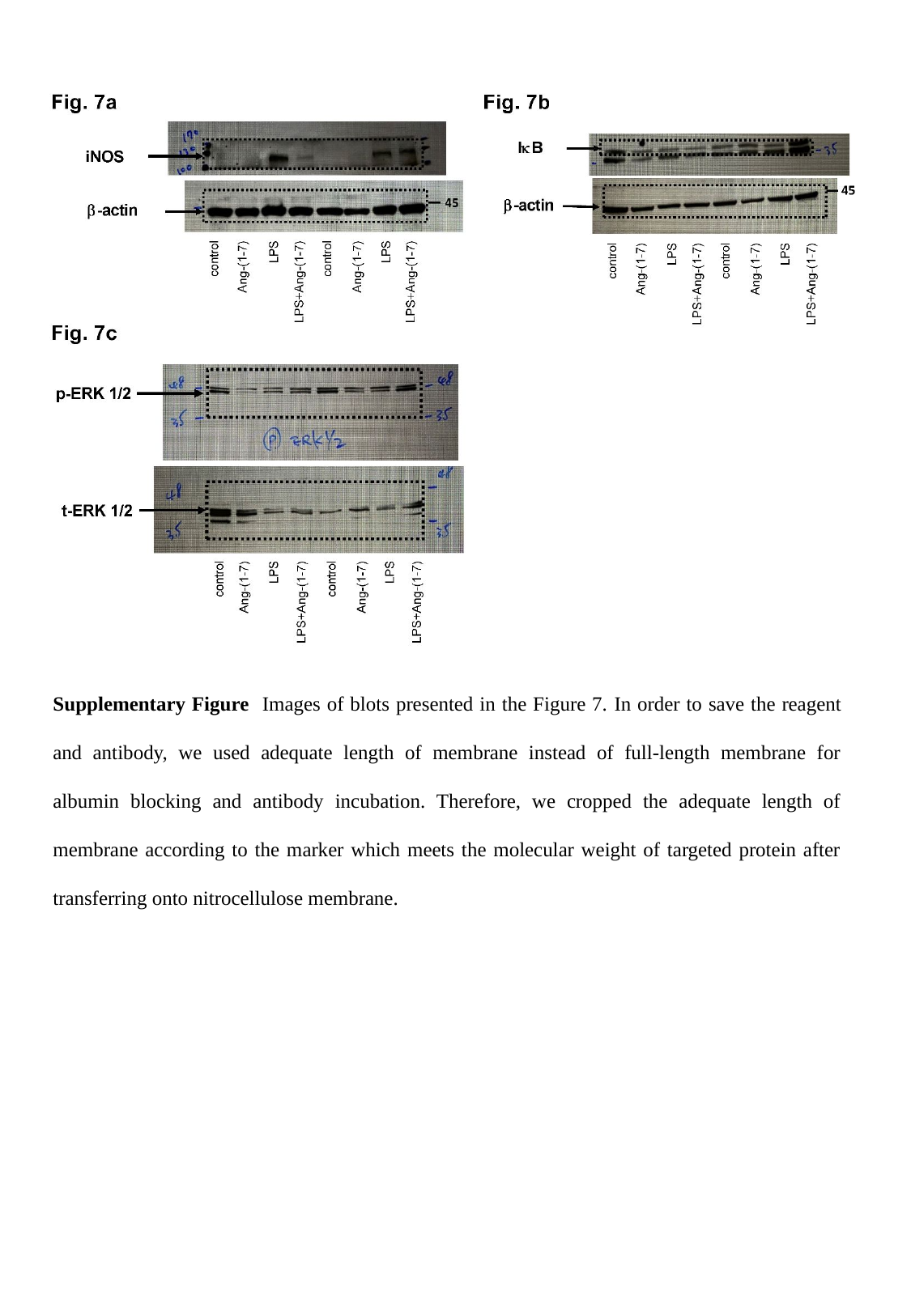

Supplementary Figure Images of blots presented in the Figure 7. In order to save the reagent and antibody, we used adequate length of membrane instead of full-length membrane for albumin blocking and antibody incubation. Therefore, we cropped the adequate length of membrane according to the marker which meets the molecular weight of targeted protein after transferring onto nitrocellulose membrane.

## Slide 4
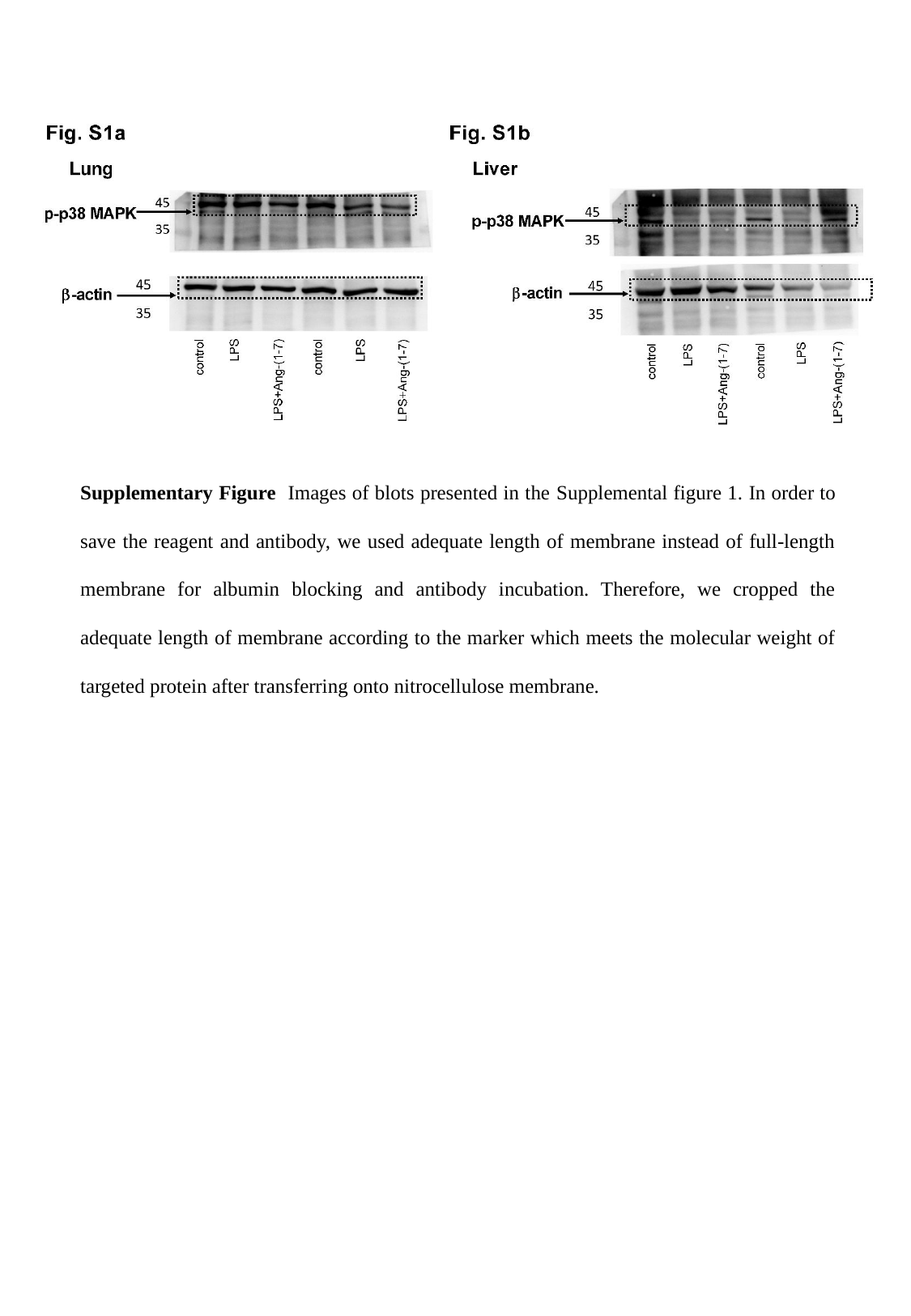

Supplementary Figure Images of blots presented in the Supplemental figure 1. In order to save the reagent and antibody, we used adequate length of membrane instead of full-length membrane for albumin blocking and antibody incubation. Therefore, we cropped the adequate length of membrane according to the marker which meets the molecular weight of targeted protein after transferring onto nitrocellulose membrane.
